# Residents’ use and perceptions of residential care facility gardens: A behaviour mapping and conversation study

**DOI:** 10.1111/opn.12283

**Published:** 2019-10-31

**Authors:** Eva Dahlkvist, Maria Engström, Annika Nilsson

**Affiliations:** ^1^ Department of Public Health and Caring Sciences Faculty of Health and Occupational Studies University of Gävle Gävle Sweden; ^2^ Department of Public Health and Caring Sciences Uppsala University Uppsala Sweden; ^3^ Nursing Department Medicine and Health College Lishui University Lishui China

**Keywords:** behaviour mapping, gardens, health, nurses, older people, residential facilities

## Abstract

**Aim:**

To describe the gardens and their use by individuals living at residential care facilities (RCFs) with high ratings on restorative values.

**Background:**

Being outdoors has been described as important to older people's well‐being. Use of outdoor gardens may increase residents’ well‐being through experiences of restorative qualities such as being away and fascination. Thus far, there has been little research on restorative experiences of gardens in the care of older people.

**Design:**

A descriptive design using behaviour mapping observations integrated with qualitative field notes and recorded conversations.

**Methods:**

A criterion sampling of two gardens (out of a total of 87) was made based on residents’ ratings of restorative values; the two with the highest values were chosen. Eleven residents at the two RCFs took part. Data were collected through behaviour mapping observations, field notes and conversations on five occasions in the respective facilities during residents’ visits to the garden.

**Results:**

The observations revealed that the main uses of the gardens were to socialise and relax. The conversations also showed that the garden stimulated residents’ senses and evoked memories from the past. These restorative values were interpreted as a sense of being away and fascination. Not having opportunities for outdoor visits was reported to result in disappointment and reduced well‐being.

**Conclusions:**

The findings showed that two basic gardens with different characteristics and views could stimulate residents’ senses and evoke memories from the past; this supports the call for residents to be able to spend time in gardens to promote their well‐being.

**Implications for practice:**

First‐line managers, nurses and healthcare staff in the care of older people should consider that regular opportunities to spend time outdoors may promote older people's well‐being through feelings of being away and fascination.

**What does this research add to existing knowledge in gerontology?**

- Previous research and present results indicate that outdoor visits can stimulate older peoples’ senses and evokes memories from the past, while insufficient opportunities can lead to poor well‐being and disappointment.
- It reveals that the gardens at RCFs were primarily used for passive stimulation in the form of socialisation and relaxation and highlights the value of providing opportunities for active stimulation.

**What are the implications of this new knowledge for nursing care with older people?**

- The results confirm previous research demonstrating the importance of taking older people's abilities and wishes into account and, to the extent possible, helping those who wish to go outdoors.
- It sheds light on staff members’ responsibility for facilitating outdoor visits and providing opportunities for restorative experiences through active or passive stimulation taking into account older people's own prioritise.

**How could the findings be used to influence policy or practice or research or education?**

- The findings are a first step in a necessarily broader, multicultural examination of the practices and experiences investigated in this article.
- The findings reveal that the outdoor environment has an important role to play in promoting older people's health and well‐being and should be utilised as an integrated part of care.
- Policymakers in health and social care need to be informed about the present and similar findings so they can improve staff members’ ability to give residents access to RCF gardens.

## 1 INTRODUCTION

Many residential care facilities (RCFs) have environments and surroundings that allow older people to be outdoors (Imamoglu, 2007). However, age‐related health problems such as multimorbidity (Akner, 2009), difficulties performing activities of daily living (ADL) (Björk et al., 2016; Roos, Silén, Skytt, & Engström, 2016), cognitive impairments (Hutsteiner, Galler, Mendoza, & Klünemann, 2013) and pain (Mamhidir et al., 2017) may prevent use of the garden and pose challenges to garden design. Well‐designed and accessible gardens, high levels of greenery and frequent visits to green outdoor spaces may promote older people's health through restorative feelings of being away and fascination (Dahlkvist et al., 2016). According to attention restoration theory (ART), a person can replenish exhausted attention capacity by visiting an environment that allow psychological distance from mental routines and demands (being away) and enable the attention to go to interesting, nice aspects of the environment (fascination). The nature offers many stimuli that may capture and engage fascination, for example to see and feel the smell of flowers, sound of leaves moving in the breeze and watch the sunset (Hartig, Kaiser, & Bowler, 1997; Kaplan, 1995). In the present study, behaviour mapping observations were used as a foreground to exploring in more detail residents’ use and perceptions of RCF gardens rated by residents as having high restorative values.

## 2 BACKGROUND

Whear et al. (2014) conducted a systematic review of studies on nine nursing homes, five special care facilities and three specialised dementia units, primarily in the United States. The quantitative studies showed decreased levels of agitation related to time spent in the garden, and the qualitative studies showed that residents used gardens for relaxation, walking, gardening and talking about design elements that could increase feelings of being away and fascination. However, only two of the seven interview studies represented residents’ views (Whear et al., 2014). Another review (quantitative and qualitative studies) (Gonzales & Kirkewold, 2013) showed that sensory gardens and horticultural activities were associated with decreased behavioural symptoms (agitation), improved sleep and reduced use of psychotropic medications in dementia care. A Swedish experimental study of nursing home residents revealed that residents’ power of concentration increased after a visit in the garden compared to resting indoors, indicating that outdoor visits were important to recover from stress and fatigue (Ottosson & Grahn, 2006). In a Finish quantitative study (Rappe, Kivelä, & Rita, 2006), nursing home residents reported a positive association between the frequency of visits to garden greenery and self‐rated health. Orr, Wagstaffe, Briscoe, and Garside (2016) systematic review of qualitative studies included people with and without dementia living in nursing homes and residential care in the United States and Europe. Their results demonstrated that garden visits were peaceful and relaxing; getting fresh air and having access to sensory impressions from nature were emphasised. Views of surrounding nature connected residents to the past and were important to their sense of being at home (Orr et al., 2016).

How gardens are used depends on design aspects, and low use may be related to poor coordination between interior and exterior spaces. A quantitative study showed that obstacles keeping residents from going out into the garden were long corridors, high thresholds, locked and heavy doors as well as hindrances in the garden itself, such as slopes, uneven ground/paths and trees/plants (Dahlkvist, Nilsson, Skovdahl, and Engström (2014). Rodiek, Lee, and Nejati (2014) found similar specific doorway problems in the form of heavy, self‐locking doors and high thresholds.

Few studies have used behaviour mapping observations to try to understand residents’ use of RCF gardens. Behaviour mapping is a method of direct observation; its main principles are place‐centred mapping and systematic behaviour samples. The goal is to observe who uses the space, how the space is used and time for use. The observer writes codes on a map to note people's ongoing activities (Ziesel, 1981). One study from the United States (Reynolds, 2016) used behaviour mapping and focus group interviews with residents at two RCFs: an assisted living facility and a continuing care retirement facility. The results showed that residents’ perceived views of nature to be important for well‐being and that the most frequent use of the garden was sitting in it, alone or together with other residents for socialising or sunning. To learn about the benefits and influence of the garden, Hernandez (2007) used behaviour mapping and interviews with staff and relatives of residents living at special care units for people with dementia in the United States. The main reason for residents’ use of the garden was simply to sit there and get some fresh air. The gardens were also rated as having positive effects on residents’ well‐being and stress recovery. Cutler and Cane (2006) used behaviour mapping, interviews with residents, staff and relatives at four nursing units in the United States to develop and describe garden design recommendations. Some of the residents reported needing more covered outdoor seating; they also mentioned insufficient access to the outdoors due to the lack of automatic door openers or staff assistance. The studies described were conducted in the United States and represent different facilities for older people. Nursing home is sometimes used as a general term for any long‐term care facility; there are various types of nursing home depending on people's needs. In Sweden, the RCFs represent nursing homes, and residents have major health problems and extensive formal care needs.

Access to and frequent use of gardens with plenty of greenery may promote older people's health, well‐being and their sense of being away and fascination. Previous research has also shown that RCF gardens can provide users with restorative and sensory experiences, socialisation, stress recovery and various activities. However, use of gardens may also depend on design aspects, and low use may be related to poor coordination between interior and exterior spaces. Thus far, little research has conducted from the residents’ perspective. An observation study with behaviour mapping, field notes and recorded conversations was conducted to better understand residents’ use of RCF gardens. The aim was to describe residents’ use and perceptions of RCF gardens that had previously been rated as high in restorative values.

## 3 METHOD

### 3.1 Design

A descriptive design was used. The study is part of a research project investigating factors related to residents’ satisfaction with and stays in RCF gardens.

### 3.2 Participants and setting

A criterion sampling method was used (Gifford, 2016), and the RCFs included in the present study are two out of totally 87 RCFs residents previously rated for their restorative value. The median value for residents’ perception of the garden includes the variables’ seasonal use, characteristics and design elements, accessibility, noise (including hustle and bustle), multisensory stimulation (Dahlkvist et al., 2014), “Being away” and “Fascination” (Dahlkvist et al., 2016), Table 1.

**Table 1:** Residents’ Perception of the Garden

Variables	Seasonal use *N* = 415^a^	Characteristics/ design elements *N* = 415 a	Accessibility *N* = 415 a	Noise *N* = 415 a	Multisensory stimulation *N* = 415 a	Being away *N* = 290^b^	Fascination *N* = 290^b^
	Median	Median	Median	Median	Median	Median	Median
RCFs (total)	2.8	2.8	3.5	3.7	2.6	6.3	5.3
Facility A	4.0	3.0	3.5	4.0	3.0	10.0	8.0
Facility B	4.0	3.5	4.0	4.0	3.0	9.0	8.0

Note

The scale for seasonal use and characteristics and design elements ranges from 0 (not at all satisfied) to 4 (very satisfied), for accessibility from 0 (agree totally) to 4 (do not agree at all), for noise from 0 (very often) to 4 (not at all) and for multisensory stimulation from 0 (seldom) to 4 (very often). The scale for being away and fascination consists of 11 points (0 = not at all to 10 (completely).

Abbreviation: RCFs, Residential care facilities.

a Dahlkvist et al. (2014).

b Dahlkvist et al. (2016).

Residents who usually visited the garden, who could walk by themselves, or wheelchair users, with or without cognitive impairments who were able to participate in a conversation during visits in the garden were asked by the manager to participate in the study. Eight women and three men (mean 86 years of age) attended (Table 2).

**Table 2:** A description of residents’ health problems according to EQ‐5D‐3L, Version 4.0, April 2011, EuroQol Group 2011 (*n* = 11)

Resident/Gender aW, bM	Age	Self‐care	Usual activities	Pain/Discomfort	Anxiety/Depression	eWalking aid/fAssistance
		EQ−5D−3L				Study‐specific question
cA1/W	90	Some problems with washing and dressing	Some problems performing usual activities	No pain or discomfort	Moderately anxious or depressed	Wheelchair
A2/W	85	Some problems with washing and dressing	Some problems performing usual activities	Moderate pain or discomfort	Moderately anxious or depressed	Wheelchair
A3/W	85	Unable to wash and dress	Some problems performing usual activities	Extreme pain or discomfort	Moderately anxious or depressed	Wheelchair electric
A4/W	85	Some problems with washing and dressing	Some problems performing usual activities	Moderate pain or discomfort	Moderately anxious or depressed	Walker
A5/W	79	Some problems with washing and dressing	Some problems performing usual activities	Moderate pain or discomfort	Moderately anxious or depressed	Walker
dB1/W	87	No problems with self‐care	No problems performing usual activities	No pain or discomfort	Moderately anxious or depressed	Wheelchair
B2/W	80	No problems with self‐care	Unable to perform usual activities	No pain or discomfort	Extremely anxious or depressed	Assistance^f^
B3/W	90	No problems with self‐care	No problems performing usual activities	No pain or discomfort	Not anxious or depressed	Assistance^f^
B4/M	94	Some problems with washing and dressing	No problems performing usual activities	No pain or discomfort	Moderately anxious or depressed	Cane
B5/M	88	Some problems with washing and dressing	Some problems performing usual activities	Moderate pain or discomfort	Moderately anxious or depressed	Wheelchair
B6/M	81	No problems with self‐care	Some problems performing usual activities	No pain or discomfort	Moderately anxious or depressed	Walker

a W = woman.

b M = male.

c Resident from Facility A.

d Resident from Facility B.

e Kind of walking aid.

f Staff assistance to go outdoors.

**Figure 1:**
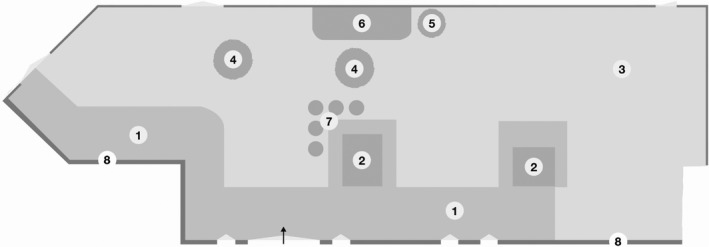
This map indicates the location and the found activity pattern for residents’ use while in the garden during observation sessions. SSCR = Sitting, socializing and relaxation. 1. Paving stones; 2. Raised garden bed; 3. Grass; 4. Tree apple; 5. Tree rowan‐berry; 6. Garden bed; 7. SSCR (Sitting, socializing and relaxation); 8. Walls

**Figure 2:**
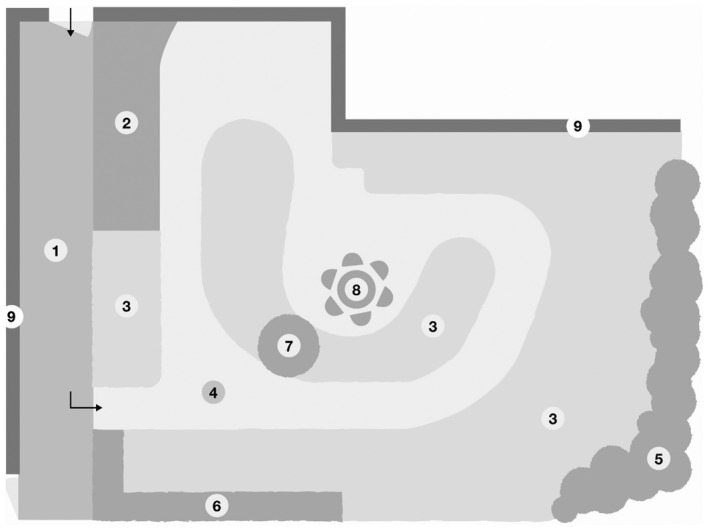
This map indicates the location and the found activity pattern for residents’ use while in the garden during observation sessions. SSCR = Sitting, socializing and relaxation. 1. Path with concrete; 2. Garden bed; 3. Grass; 4. Gravels; 5. Hedge mountain currant; 6. Hedge syren; 7. Tree cherry; 8. SSCR (Sitting, socializing and relaxation); 9. Walls

One of the RCFs (Facility A) is a multi‐story building with 4 floors and 32 apartments for residents in need of considerable formal care. Facility A is situated in an industrial municipality, near the city centre with the nature surrounding. On the first floor, a therapy/activity centre is open Monday to Friday, staffed by an occupational therapist in addition to regular staff at the facility. The therapy/activity centre has a door through which residents can enter the garden (Figure 1). The other RCF (Facility B) is a one‐story building with places for 23 residents in need of formal care due to cognitive impairments. Facility B is situated on a prominence in an urban area in central Sweden with the nature surrounding; it also has a garden intended to stimulate residents’ senses. From the garden, one can see an adjacent road, a private house and mountain views. A common patio door leads from a corridor out into the garden (Figure 2). At both facilities, the staff consists of a manager, Registered Nurses, licensed practical nurses and nursing assistants.

### 3.3 Data collection

The managers were informed that their RCF garden's restorative value had been rated high, informed about the present study and invited to participate. Residents and the relatives of persons with cognitive impairments received written and oral information from the manager at each facility. A map was constructed for each garden and marked with codes (letters and numbers) for different areas and existing design features (Figures 1 and 2).

### 3.4 Behaviour mapping, field notes and conversations

Data were collected in June and July 2012 using behaviour mapping observations, field notes and taped conversations on five occasions at the respective facilities. Behaviour mapping is a less intrusive method of direct observation; its main principles are place‐centred mapping and systematic behaviour samples. At predetermined intervals, the observer notes the activity of people within a given area. By systematically using sketches for a space and unique codes for each participant, the goal is to observe who uses the space, how it is used and time for use. This method is based on previous studies conducted by environmental designers (Ziesel, 1981) and has been used in previous studies on older people (Cutler & Kane, 2006; Hernandez, 2007; Reynolds, 2016).

Observations were systematically performed in the gardens at various times of day (10:00–10:45 a.m., 10:00–11:20 a.m., 13:00–13:45 p.m. and 13:00–14:20 p.m.), and each occasion varied between 45 to 80 min (mean = 63 min). During the observations, field notes were taken concerning who was in each area, what they did there and for how long. After having observed the residents in the garden, a pattern emerged. For instance, if a participant visits the same areas routinely day after day, a pattern would emerge revealing the same “markings on the map,” repeatedly.

The audio‐recorded conversations lasted between 20–40 min and were performed with the participants in the garden in conjunction with the observation. These were intended to complement the observations and discover whether there was anything that could not be answered by the observations. Because the conversations took place in the garden, other residents were also there when the conversations took place. Both the researcher (ED) and the participants had Swedish as native speech. Starting from the four topics in the conversation guide, the participants were encouraged to speak freely about their use of and preconditions for visiting the garden (Table 3).

**Table 3:** Question areas

Why are you out in the garden today and what makes you want to be outside?
What parts of the garden do you usually visit?
What do you usually do in the garden, by yourself or with others?
Do you experience any difficulties associated with being in the garden? (e.g., getting around, places to sit, cover from the sun, tall plants, the ground, visibility, influence of weather, etc.)
Complementary questions were asked when further clarification was needed, for example: Did I understand you correctly? Can you tell me more about that?

### 3.5 Data analysis

All behaviour mapping observations of the respective occasions were recorded on a series of coded place‐centred maps to represent participants’ garden use during intervals and to locate particular areas of the garden as well as types of activities they were observed to be engaged in during the observation periods. The occasions for each observation were then composited into a single place‐centred map to obtain a pattern for participants’ garden use. Thereafter, the field notes were analysed to confirm whether or not they were consistent with the observations. Content analysis was used to analyse the conversations (Patton, 2015). All conversations were audiotaped, transcribed verbatim and listened to, and the transcripts were read repeatedly so as to achieve an understanding of and become familiar with the text. Meaning units were created, condensed and coded—using one or two words that expressed the core of each meaning unit—and then categorised. Data were compared for similarities and differences, and the analysis process consisted of back‐and‐forth movements among the whole texts. During the analysis process, all authors discussed different steps to reach a broad consensus, thus increasing the study's credibility and dependability.

### 3.6 Research considerations

Residents and relatives were informed about the study, voluntary participation and that their decision would not in any way influence their future care. Written informed consent was obtained from the residents. Prior to each conversation, the observer introduced and explained the intention of the conversations, and repeated the information on informed consent. The Regional Ethical Review Board approved study (Reg. no.: 2011/139).

## 4 RESULTS

Maps and texts for the respective facilities are used to present the results from the behaviour mapping observations, field notes and conversations. The residents are coded with letters and numbers. In Facility A, they are represented as A1 to A5, and in Facility B as B1 to B6. Interview quotes provided to support the residents’ descriptions and the credibility of the results. During the study period, the weather was usually sunny, though sometimes cloudy and windy.

### 4.1 Behaviour mapping observations and conversations with the residents

The residents’ descriptions were primarily consistent with the observations, field notes and the marked codes on the maps. The pattern on the maps revealed what garden spaces the residents typically visited by themselves or with others. The conversations described the residents’ perceptions of and prerequisites for visiting the garden.

The general pattern from the observations in Facility A showed that the residents were mainly positioned by the staff in the same spaces in the garden. For the most part, they sat close together in a row, adjacent to one of the two raised garden beds near the facility entrance door (Figure 1). Due to their dependence on staff assistance, it was less common for them to go to other places in the garden by themselves. However, the conversations also revealed that a few residents usually visited other areas of the garden: “*I usually roll around looking at things but mostly I sit by the embankment up there”* (A3). The observations and conversations combined showed that the main use of the garden was to socialise and relax. Socialisation usually consisted of conversations between residents about the day's weather, garden greenery and, for example, the flowers growing in a raised bed, while relaxation consisted of just sitting and relaxing and/or getting some sleep in the sun. Besides the described general pattern, on one occasion two residents were weeding one of the raised garden beds.

The observations at Facility B revealed a general pattern similar to that at Facility A. The staff typically sat residents who needed assistance around a table in the middle of the garden (Figure 2). The main uses of the garden were for socialisation and relaxation. Socialisation usually consisted of conversations between three of the residents, who used to sit in the sun and talk to each other about the weather and what snacks they would like. Relaxation was simply a matter of sitting and relaxing, and/or sunbathing. There were also occasions when they sat quietly and looked at the view from the facility as well as at the adjacent road. The observations also showed that, on two occasions, the residents were able to socialise with care dogs. The conversations in Facility B also revealed that a few residents mentioned typically sitting at the table in the garden, sometimes by themselves: “*Mostly I sit at the table here”* (B1); “*Sometimes I sit by myself, but I don't have any special places where I like to sit”* (B6)*.*

The conversations at both Facility A and B showed that the main use of the garden was for common meals and socialising with other residents: “*I like sitting around the table, then there's coffee and lots of friends are here”* (B1); “*I usually don't talk, but at least I’m not alone when I’m out here*” (A1). Other activities commonly mentioned related to relaxing: “*I usually don't do anything at all except get some sun and drink coffee”* (B3); “*I sat and rested, and it was nice to relax; I fell asleep. You get tired sitting still and not doing anything”* (A1). Some residents said that activities in the garden could strengthen one's sense of having skills: “*Well, I dug out the bad plants in the growing beds over there”* (A3); “*I did some raking and just had fun, you get to show that you know how to do something”* (B2). One of the residents in Facility A said that she talks to the children at the kindergarten near the fence on the facility property: “*I usually talk to the little children there; they come over and say hi, they usually give me flowers”* (A5).

The conversations also demonstrated that one of the residents did not want to do anything special and longed to return to his/her former home, while another declared that the most important thing was having the opportunity to go outdoors: “*But you know, I don't want to do anything special, I want to go home, to our home”* (B3); “*I’m satisfied with the garden, I think it's so nice just to get outside and get some air now and then”* (A3). In contrast, there were residents who said they wished to do activities: “*I’d like to get down on my knees and pull up all the weeds. At home I cleaned up between the plants, dug out all the weeds between them”* (A2); “*If there was something to do I’d like to grow potatoes”* (B6)*.*

### 4.2 The residents’ perceptions during garden visits

This category describes what emerged from the conversations concerning the residents’ described perceptions during their visits in the garden, what they usually look at, think about and the conceivable consequences of not having opportunities to go outdoors.

Several of the residents described memories related to gardens, relatives and pets from the past: “*Just think when we were home and I sat behind the cabin where the sun shone from noon to evening. My mother was crazy about plants. And we had a cat”* (B3); “*It reminds me a lot of the garden we had when my husband was alive. We had a greenhouse with tomatoes and cucumbers; we ate vegetables until we almost exploded”* (A2). Some residents also talked about the garden's importance for stimulating their senses and their fascination with seeing and smelling the flowers and seeing pets: “*I think about the lilacs behind us here, they're so lovely! I can't get too close, but I can smell them from here”* (B1); “*I really like the kitty cat here, I had one once, he was steel‐grey. Cats are nice”* (A2); “I got small wild bleeding heart plants from my sister‐in‐law who had a cabin on a mountain. She'd planted them in every little hollow and it was so pretty*” (A1).*

Some statements showed that residents experienced decreased well‐being when they were unable to be outdoors: “*I don't just want to sit inside, then I don't feel good, I get tired. I’d rather be outside, I like being outside”* (B1)*.* Others revealed that not being able to go outside could lead to disappointment or even the risk of becoming hysterical: “*I get disappointed if I can't get outside every day; I’ve done it my whole life”* (B3): “*If I can't be outside and get some sun I become hysterical”* (B1)*.*

### 4.3 Prerequisites for garden visits

This category describes prerequisites for residents visiting the garden; it consists of two sub‐topics: *weather conditions* and *obstacles to visiting the garden.*

#### 4.3.1 Weather conditions

At both facilities, weather conditions were often a prerequisite for residents wanting to go outdoors. Thus, experiencing sunny weather and fresh air was the main reason for outdoor visits and their feelings of well‐being: “*Ever since I came here I’ve tried to take advantage of every ray of sun there's been”* (A3); “*Yes, it's the sun I enjoy, my best ever free friend”* (B1)*.* In most cases, the conversations gave no sign that the weather ever prevented residents from to going outdoors: “*You have to be thankful for the weather you get.”* (B1); “*I can go out in any kind of weather. As long as you dress right, no such things as bad weather, only bad clothes”* (A5). However, a few residents felt that some weather conditions were difficult: “*I don't want to sit in the hot sun, I sit in the shade in the summer, otherwise I get a headache”* (A1); “*It's so windy it's like you feel now something's coming. Well, that's how it is, hope I can manage it”* (B2)*.*

#### 4.3.2 Obstacles to visiting the garden

The observations and field notes showed that the residents were typically dependent on staff assistance to go outdoors. During the conversations, several of the residents also mentioned that: “*They take you out when you can't do it yourself, it's great. On the weekends you better hope someone comes to visit so you can go outside, otherwise the days are long”* (A1); “*Yes, they took me out. Really I’d like to be outside all the time”* (B1)*.* Some of the residents mentioned the need for help to find their way out into the garden: “*Sometimes I can't find my way out into the garden, I walk around and around. Well, it makes you sad when you get lost. The halls are so long, I call them runways”* (A4). Furthermore, the statements revealed that obstacles in the garden threatened their sense of security: “*I have a hard time moving forward with this chair. In the grass over there you have to go up and down. I think, oh God, I’m gonna tip over here soon. It's an awful feeling, I really don't want to go up there”* (A3) “*It's not great, it's hard to walk on the gravel with my walker” * (B6)*.*

## 5 DISCUSSION

The main results showed that two basic gardens with different characteristics and views could stimulate residents’ senses and evoke memories from the past. The common uses were for socialising and relaxation, while specific activities occurred to a limited extent. Some residents connected not having opportunities for outdoor visits to feelings of reduced well‐being and disappointment. However, the residents needed staff assistance due to their own preconditions and/or the presence of design‐related obstacles on the way out to and in the garden.

There were residents who reported being fascinated by seeing and smelling flowers and seeing pets in the garden. One resident mentioned the views of mountains and a nearby kindergarten and enjoyed looking at and talking to the children. Some of the residents mentioned wanting to cultivate plants. However, the observations revealed few ongoing activities and that the staff were more active with cultivation in the facility nearby the kindergarten because an occupational therapist worked there daily. This might have contributed to the residents’ high restorative value for this facility. These findings could be understood in relation to ART (Hartig et al., 1997; Kaplan, 1995) and its concepts being away and fascination—feelings nature has the ability to elicit in individuals. Being away refers to distancing oneself from routine mental contents and may have contributed to residents’ experiences of feeling relaxed during garden visits. Appreciating views of children and nature, flowers and pets in the garden, the desire to cultivate and cultivation opportunities may relate to residents’ sense that this kind of content is fascinating, but not demanding (Hartig et al., 1997; Kaplan, 1995). Kaplan (1995) stated that a restorative environment must provide enough for a person to see and experience “so that it takes up a substantial portion of the available room in one's brain” (pp 173). Thus, RCF staff should facilitate outdoor visits with possibilities for restorative experiences through active or passive stimulation of residents’ senses and by offering interactions with, for example plants, flowers and pets.

The observations and conversations showed that the most common uses of the gardens were to socialise and relax. Relaxation was described as, for example, doing nothing at all besides sunbathing and taking a short nap. In line with this result, two reviews (Orr et al., 2016; Whear et al., 2014) found that residents felt use of the garden could be peaceful and relaxing. Studies have also revealed that exposure to and visits in gardens with considerable greenery may provide restorative effects and promote health among older people living in RCFs Rappe et al. (2006) and Dahlkvist et al. (2016). According to ART, a restorative environment gives the visitor an opportunity to experience a sense of being away without having to engage in routine everyday tasks and demands (Kaplan, 1995). For older people suffering from complex health problems, socialisation and relaxation in a garden—a restorative outdoor environment—might promote health, protect against fatigue, and mitigate stress and demanding situations.

The overall pattern revealed through the observations was that garden activities occurred to a limited extent. This might be because the residents themselves had different preferences and needs for outdoor visits. It might also be because there were no garden‐related routines or organised activities. Hernandez (2007) stated that active stimulation refers to *doing* and taking part in activities, for example socialising and picking flowers, while passive refers to *being* in the garden and experiencing different sensory stimulations, for example birdsong and views of nature (Hernandez, 2007). ART emphasises that an environment is restorative if it supports what an individual wants to do (active stimulation) and if it is rich in fascinating features (passive stimulation) (Kaplan, 1995).

According to the residents, not having opportunities to be outdoors could lead to reduced well‐being and disappointment. However, the observations and conversations revealed several prerequisites for residents to use and have restorative experiences in the garden. One was the residents’ own physical preconditions, as they needed staff assistance or encouragement to visit the garden. In line with this, qualitative studies (Grant & Wineman, 2007; Kearney & Winterbottom, 2006) and one quantitative study (Rappe & Kivelä, 2005) have found additional prerequisites for getting outdoors: staff willingness and attitudes towards encouraging residents to go outdoors.

In our study, and as described by Bengtsson and Carlsson (2013), another prerequisite was the weather conditions, which sometimes affected residents’ willingness to go out. In accordance with this, Dahlkvist et al. (2014) found statistically significant differences between residents’ satisfaction with outdoor stays during different seasons. However, in our study, some residents recounted the popular phrase that there is “no bad weather, only bad clothes.” The staff should therefore facilitate possibilities for using the garden in different weather conditions.

Another prerequisite was obstacles on the way out into the garden. This is consistent with research showing that design characterised by poor coordination between interior and exterior spaces may reduce the accessibility and use of the garden. A survey and interview study found that the main obstacles to outdoor use were thresholds and self‐locking doors (Rodiek et al., 2014). Gonzales and Kirkevold (2016) examined the design characteristics of sensory gardens using a cross‐sectional web‐based survey aimed at RCF managers. The results demonstrated that inability to open doors autonomously hindered optimal garden use by residents. Moreover, Dahlkvist et al. (2014) described characteristics and design elements from a management perspective and found that, for residents, going outdoors was troublesome due to long corridors, stairs, locked and heavy doors.

Other prerequisites concerned obstacles in the garden that made it difficult for residents to enjoy restorative experiences and move around in the garden. This threatened their sense of safety and security. For example, one participant was afraid that his/her wheelchair would tip backwards, while another talked about the risk that his/her walker would get stuck in the gravel. According to Bengtsson and Grahn (2014), some important qualities are required to feel comfortable in the outdoor environment at RCFs. For example, level, slip‐resistant, glare‐free walking surfaces help to minimise falls and walkways and are designed to support balance and coordination (Bengtsson & Grahn, 2014). These findings are supported by Dahlkvist et al.’s (2014) study, which showed that residents were more satisfied with the garden design when fewer obstacles were present.

### 5.1 Methodological considerations

The predetermined criterion was to include RCF gardens based on a previous study in which residents rated a total of 87 gardens, of which the two with the highest restorative values were chosen. The sampling method enabled examination of gardens that residents felt had high restorative qualities. One strength of the study may be the use of behaviour mapping observations, which allow the researcher to directly observe and record what occurs in context. Use of this method also has limitations. Place‐centred mapping and systematic behaviour samples may be too intrusive and reactivity is a problem, which means that people are aware they are being observed. To counteract intrusiveness and reactivity, the researcher therefore attempted to blend into the surroundings as much as possible (Gifford, 2016). To increase the credibility and dependability of the results, different considerations were taken into account. Method triangulation was used, meaning that different data collection methods—observations and conversations with field notes—were employed. The conversations were carried out in conjunction with the participants’ outdoor visits in the garden, which may be seen as an advantage. It could also be seen as a disadvantage, because other residents in the garden could disturb the participants. However, they were performed outdoors to get closer to participants’ perceptions in the environment under study and to support their memory. All authors discussed the different steps to achieve agreement during the analysis process. Data were collected on five occasions at the respective facilities in conjunction with residents’ visits to the garden. This can be considered a sufficiently long period for the researcher to learn about the culture of the studied group and to avoid misunderstandings and distortions of the observations. The present study extends earlier research by Dahlkvist et al. (2014), Dahlkvist et al. (2016), which revealed that greenery index (e.g. trees, shrubs, lawns, flowers, raised garden beds, and water‐related elements) is indirectly related to individuals’ experiences of health, mediated by restorative values (Dahlkvist et al., 2016). The findings of the present study are the first step in a necessarily broader, multicultural examination of the practices and experiences investigated in this article. The description of resident's demographic characteristics, details about data collection and the analysis process should enable transferability to similar contexts.

## 6 CONCLUSION

The behaviour mapping observations and conversations in two gardens with different characteristics showed that residents primarily used the gardens for socialising and relaxation. The conversations uncovered that outdoor visits are important to residents’ socialisation and restorative experiences in the form of relaxation, stimulation of the senses and memories from the past. Several factors were found to either promote or hinder going outdoors.

## CONFLICT OF INTEREST

No conflict of interest has been declared by the authors.

1 **Implications for practice**

- Health‐care staff should have the knowledge and education about how the outdoor environment can promote older people’s health and well‐being.
- The first‐line managers should create possibilities for the health‐care staff so older people can spend more time outdoors.
- To spend time in the outdoor environment can promote older people’s health and well‐being and should therefore be integrated as a daily routine in care.
